# Supplementary material for: Pyruvate Dehydrogenase Kinase 1 inhibition mediated oxidative phosphorylation enhancement in cartilage promotes osteoarthritis progression
Source: BMC Musculoskelet Disord. 2023 Jul 20;24:597. doi: 10.1186/s12891-023-06585-6 (PMC10357736; doi:10.1186/s12891-023-06585-6)
Supplement: Supplementary file 1 — Additional file 1. [file 12891_2023_6585_MOESM1_ESM.zip › KEGG.Gsea.1653622432464/KEGG_ETHER_LIPID_METABOLISM.html]

Details for gene set KEGG\_ETHER\_LIPID\_METABOLISM[GSEA]

|  || Dataset | 55457\_collapsed\_to\_symbols.cls.cls #OA\_versus\_normal.cls.cls #OA\_versus\_normal\_repos |
| Phenotype | cls.cls#OA\_versus\_normal\_repos |
| Upregulated in class | normal |
| GeneSet | KEGG\_ETHER\_LIPID\_METABOLISM |
| Enrichment Score (ES) | -0.19418068 |
| Normalized Enrichment Score (NES) | -0.6397807 |
| Nominal p-value | 0.9440559 |
| FDR q-value | 1.0 |
| FWER p-Value | 1.0 |
Table: GSEA Results Summary

  

Fig 1: Enrichment plot: KEGG\_ETHER\_LIPID\_METABOLISM      
 Profile of the Running ES Score & Positions of GeneSet Members on the Rank Ordered List

  

| SYMBOL | TITLE | RANK IN GENE LIST | RANK METRIC SCORE | RUNNING ES | CORE ENRICHMENT || 1 | PLA2G12A | phospholipase A2 group XIIA [Source:HGNC Symbol;Acc:HGNC:18554] | 600 | 0.623 | 0.0401 | No |
| 2 | PLPP1 | phospholipid phosphatase 1 [Source:HGNC Symbol;Acc:HGNC:9228] | 1109 | 0.494 | 0.0694 | No |
| 3 | AGPS | alkylglycerone phosphate synthase [Source:HGNC Symbol;Acc:HGNC:327] | 1727 | 0.394 | 0.0763 | No |
| 4 | PAFAH1B1 | platelet activating factor acetylhydrolase 1b regulatory subunit 1 [Source:HGNC Symbol;Acc:HGNC:8574] | 1874 | 0.376 | 0.1173 | No |
| 5 | PLA2G4A | phospholipase A2 group IVA [Source:HGNC Symbol;Acc:HGNC:9035] | 2656 | 0.284 | 0.0962 | No |
| 6 | PAFAH1B2 | platelet activating factor acetylhydrolase 1b catalytic subunit 2 [Source:HGNC Symbol;Acc:HGNC:8575] | 3151 | 0.235 | 0.0905 | No |
| 7 | PLA2G10 | phospholipase A2 group X [Source:HGNC Symbol;Acc:HGNC:9029] | 3315 | 0.220 | 0.1085 | No |
| 8 | PLA2G3 | phospholipase A2 group III [Source:HGNC Symbol;Acc:HGNC:17934] | 3648 | 0.190 | 0.1092 | No |
| 9 | CHPT1 | choline phosphotransferase 1 [Source:HGNC Symbol;Acc:HGNC:17852] | 3911 | 0.163 | 0.1116 | No |
| 10 | PAFAH2 | platelet activating factor acetylhydrolase 2 [Source:HGNC Symbol;Acc:HGNC:8579] | 4446 | 0.118 | 0.0864 | No |
| 11 | LPCAT4 | lysophosphatidylcholine acyltransferase 4 [Source:HGNC Symbol;Acc:HGNC:30059] | 4615 | 0.104 | 0.0878 | No |
| 12 | PLA2G2D | phospholipase A2 group IID [Source:HGNC Symbol;Acc:HGNC:9033] | 5056 | 0.069 | 0.0632 | No |
| 13 | LPCAT1 | lysophosphatidylcholine acyltransferase 1 [Source:HGNC Symbol;Acc:HGNC:25718] | 5957 | -0.002 | -0.0064 | No |
| 14 | PAFAH1B3 | platelet activating factor acetylhydrolase 1b catalytic subunit 3 [Source:HGNC Symbol;Acc:HGNC:8576] | 6060 | -0.009 | -0.0131 | No |
| 15 | PLA2G5 | phospholipase A2 group V [Source:HGNC Symbol;Acc:HGNC:9038] | 6772 | -0.061 | -0.0599 | No |
| 16 | PLA2G2E | phospholipase A2 group IIE [Source:HGNC Symbol;Acc:HGNC:13414] | 7486 | -0.117 | -0.0990 | No |
| 17 | PLA2G1B | phospholipase A2 group IB [Source:HGNC Symbol;Acc:HGNC:9030] | 8232 | -0.173 | -0.1327 | No |
| 18 | ENPP2 | ectonucleotide pyrophosphatase/phosphodiesterase 2 [Source:HGNC Symbol;Acc:HGNC:3357] | 8442 | -0.189 | -0.1226 | No |
| 19 | PLD1 | phospholipase D1 [Source:HGNC Symbol;Acc:HGNC:9067] | 9365 | -0.261 | -0.1578 | Yes |
| 20 | PLA2G7 | phospholipase A2 group VII [Source:HGNC Symbol;Acc:HGNC:9040] | 9467 | -0.271 | -0.1280 | Yes |
| 21 | PLD2 | phospholipase D2 [Source:HGNC Symbol;Acc:HGNC:9068] | 9615 | -0.284 | -0.0998 | Yes |
| 22 | PLPP3 | phospholipid phosphatase 3 [Source:HGNC Symbol;Acc:HGNC:9229] | 9906 | -0.312 | -0.0789 | Yes |
| 23 | PLA2G2A | phospholipase A2 group IIA [Source:HGNC Symbol;Acc:HGNC:9031] | 10152 | -0.336 | -0.0510 | Yes |
| 24 | PLA2G2F | phospholipase A2 group IIF [Source:HGNC Symbol;Acc:HGNC:30040] | 10628 | -0.386 | -0.0342 | Yes |
| 25 | PLA2G6 | phospholipase A2 group VI [Source:HGNC Symbol;Acc:HGNC:9039] | 11128 | -0.446 | -0.0109 | Yes |
| 26 | PLPP2 | phospholipid phosphatase 2 [Source:HGNC Symbol;Acc:HGNC:9230] | 11160 | -0.452 | 0.0496 | Yes |
| 27 | JMJD7-PLA2G4B | JMJD7-PLA2G4B readthrough [Source:HGNC Symbol;Acc:HGNC:34449] | 12076 | -0.614 | 0.0640 | Yes |
Table: GSEA details [plain text format]

  

Fig 2: KEGG\_ETHER\_LIPID\_METABOLISM      
 Blue-Pink O' Gram in the Space of the Analyzed GeneSet

  

Fig 3: KEGG\_ETHER\_LIPID\_METABOLISM: Random ES distribution      
 Gene set null distribution of ES for **KEGG\_ETHER\_LIPID\_METABOLISM**
